# P-bRS: A Physarum-Based Routing Scheme for Wireless Sensor Networks

**DOI:** 10.1155/2014/531032

**Published:** 2014-02-05

**Authors:** Mingchuan Zhang, Wangyang Wei, Ruijuan Zheng, Qingtao Wu

**Affiliations:** Information Engineering College, Henan University of Science and Technology, Luoyang 471023, China

## Abstract

Routing in wireless sensor networks (WSNs) is an extremely challenging issue due to the features of WSNs. Inspired by the large and single-celled amoeboid organism, *slime mold Physarum polycephalum*, we establish a novel selecting next hop model (SNH). Based on this model, we present a novel Physarum-based routing scheme (P-bRS) for WSNs to balance routing efficiency and energy equilibrium. In P-bRS, a sensor node can choose the proper next hop by using SNH which comprehensively considers the distance, energy residue, and location of the next hop. The simulation results show how P-bRS can achieve the effective trade-off between routing efficiency and energy equilibrium compared to two famous algorithms.

## 1. Introduction

Wireless sensor networks (WSNs) are a class of wireless ad hoc networks which consist of a set of sensor nodes and aim at several applications, such as industrial sensing and control and environment monitoring [1]. Each sensor node is a low-cost, short range wireless transceiver typically equipped with a low-computation processor and a battery operated power supply. Under many cases, sensors need to work without battery replacement for several years. Thus, there are two questions needing to be considered. One is how to achieve the energy balance of sensor nodes to avoid the emergence of energy hole which commonly occurs around the *Sink*, since the data traffic follows a many-to-one communication pattern and nodes nearer the *Sink* have to take heavier traffic load. The other is how to obtain high routing efficiency under multihop transmission circumstance, since WSNs can contain hundreds of such low-cost sensor nodes. Therefore, designing such networks should primarily focus on both routing efficiency and energy equilibrium in terms of trade-off.

Location-aware routing protocols seem to possess high routing efficiency. However, there are two extremes in location-aware routing, the greedy strategy and the robust strategy. Greedy strategies may suffer failures to route packets to destination, while robust strategies need very high flooding rates to ensure reliability and rapid delivery of data. Thus, many location-aware routing protocols are mostly to propose methods to overcome the mentioned drawbacks. GPSR [2] is a famous greedy routing protocol, which makes greedy forwarding decisions using only information about a router's immediate neighbors. Sivrikaya et al. [3] propose randomized routing based on Markov chains to balance the load and routing performance. Kuhn et al. [4] utilize face (or perimeter) routing to go around voids in the topology. Bai et al. [5] present a routing algorithm which routes the connections in a manner that link failure does not shut down the entire stream but allows a continuing flow for a significant portion of the traffic along multiple paths to address the issues of reliability and energy efficiency. Trajcevski et al. [6] present heuristic approaches to relieve some of the routing load of the boundary nodes of energy holes in location-aware WSNs. Wang and Syue [7] propose a relay selection protocol based on geographical information, in which multihop transmission is realized by concatenation of single cluster-to-cluster hops.

The energy-aware routing attracts more attention of researcher than location-aware routing for the significance of energy. For maximizing the network lifetime, Rao and Fapojuwo [8] present a battery-aware distributed clustering and routing protocol which incorporates the state of the battery's remaining charge and health parameters in computing the charge utility metric at each cluster formation round. Trajcevski et al. [9] construct a data aggregation tree that minimizes the total energy cost of data transmission. By allowing the battery to rest for certain duration, without being subjected to heavy loads, Chau et al. [10] consider that a portion of the lost charge can be recovered due to the battery's recovery effect and present a battery model. A battery-aware power allocation model was studied in [11] for a single-hop transmission scheme to balance the network energy consumption based on the nonlinear battery parameters proposed in [12].

In recent years, bio-inspired technology has been concerned by researchers [13–15]. We draw the inspiration from the slime mold *Physarum polycephalum* which is a large and single-celled amoeboid organism. Nakagaki et al. [16] validate physarum which is apparently able to solve shortest path problems by constructing a maze. Tero et al. [17] use physarum forms of a network with comparable efficiency, fault tolerance, and cost to those of Tokyo rail system. Tero et al. [18] propose a mathematical model for the behavior of physarum and argue extensively that the model is perfect. We migrate the physarum foraging model to wireless networks to develop physarum-based routing algorithms through dimensionless analogy analysis [19, 20].

Based on our prior works [19–23], this paper focuses on how to choose the proper next hops to transmit data to the *Sink* in thinking of both routing efficiency and energy equilibrium, which is partially same to GEAR [24], other than only depending on the remaining energy [25]. The rest of this paper is organized as follows.  Section 2 formulates the bio-inspired model.  Section 3 proposes the P-bRS routing scheme.  Section 4 evaluates our P-bRS by simulations. Finally, the conclusion is presented.

## 2. System Models

### 2.1. Typical WSNs Scenario

This paper considers large multihop WSNs which consist of *n* static sensor nodes and a mobile *Sink* node, and the sensor nodes are distributed uniformly in a two-dimensional space. We assume that (1) all sensor nodes are aware of their locations, which may be achieved through GPS receivers or other methods; (2) each sensor node is aware of its energy residue; and (3) the *Sink* node moves along a certain orbit in the field and broadcasts periodically its current positions.

**Figure 1:**
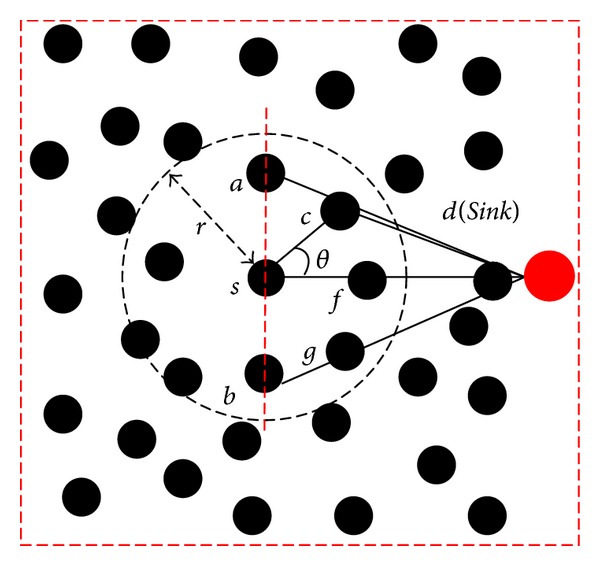
An example of WSNs' topology.

In WSNs, each node *i* has a fixed circular transmission range *r* which determines the set of sensors in which each node can communicate with node *i* in one hop. We abstract such WSNs using a graph *G* = (*V*, *E*), where each node *v* ∈ *V* represents a sensor and each edge *e* ∈ *E* represents the existence of one-hop wireless link between two sensors. An example of WSNs' topology is shown in Figure 1.

The transmission range of *s* is drawn as a dashed circle whose radius is *r* and center is *s*. We call the angle *θ* to be the angle of deviation, which represents a measurement of the next hop deviating from the *Sink* *d*. The Euclidean distance of any two nodes and the angle *θ* can be calculated following (1) and (2), respectively. Consider

(1) $$\begin{array}{c} L_{ij}=\sqrt{{\left(x_{i}-x_{j}\right)}^{2}+{\left(y_{i}-y_{j}\right)}^{2}}, \end{array}$$

(2) $$\begin{array}{c} \theta =\text{arccos}\frac{L_{sc}^{2}+L_{sd}^{2}-L_{cd}^{2}}{2\times L_{sc}\times L_{sd}}, \end{array}$$

where (*x*_*i*_, *y*_*i*_) and (*x*_*j*_, *y*_*j*_) are the coordinates of nodes *i* and *j*, respectively.

If the node *s* needs to transmit data to the *Sink* *d*, it will select its next hop in the dashed circle. However, the nodes in semicircle far from the *Sink* are apparently inappropriate to be chosen as the next hop. Usually, we choose the next hop in the semicircle nearer to the *Sink*. Obviously, the smaller the angle *θ* is, the closer the next hop is to the *Sink*. Therefore, we are apt to choose the node whose *θ* is smaller as the next hop. In order to simplify discussion, we define the *N*_*s*_, *N*_*s*_^*F*^, and *N*_*s*_^*N*^ as the sets of neighbors (the nodes in the dash circle in Figure 1), far neighbors (the nodes in the left dash circle in Figure 1), and near neighbors (the nodes in the right dash circle in Figure 1), of node *s*, respectively, where *N*_*s*_, *N*_*s*_^*F*^, and *N*_*s*_^*N*^ satisfy *N*_*s*_ = *N*_*s*_^*F*^ ∪ *N*_*s*_^*N*^ and *N*_*s*_^*L*^∩*N*_*s*_^*R*^ = *∅*.

In addition, acquiring energy residues of neighbors is important for choosing next hop to balance the energy of sensor's nodes. We think of the basic theory of wireless transmission combing with Figure 1. If node *s* transmits a group of data to *c*, all of the nodes in *N*_*s*_ would receive the wireless radio and check the packet header. The node *c* matches the field *Destination* and receives the packet. Other nodes mismatch the field *Destination*, then ignore the packet, and go on sleeping.

In order to acquire the energy residue of neighbors, we add a new field *Energy*_*Residue* to the packet header. When node *s* transmits a group of data to *c*, all of the nodes in *N*_*s*_ extract the fields of *Source* and *Energy*_*Residue* from the packet header and save *Energy*_*Residue* in local memory according to field *Source*. Then, the node *c* matches the field *Destination* and receives the packet. Other nodes mismatch the *Destination*, then ignore the packet, and go on sleeping. Since each node needs to listen in real time to every packet and try to match its field *Destination*, only adding an operation of saving *Energy*_*Residue* would not add a considerable effect on energy consumption. Therefore, we neglect the cost of acquiring energy residue of neighbors.

**Figure 2:**
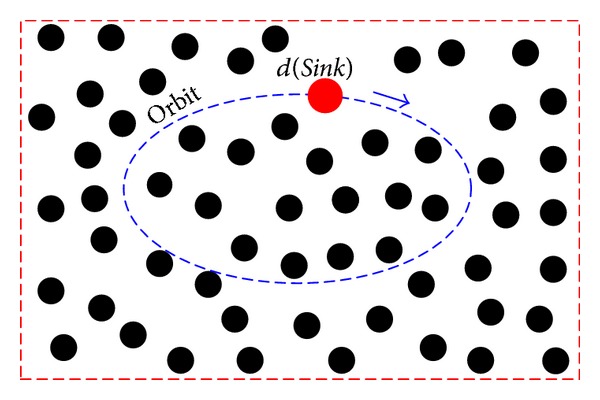
The orbit of the *Sink* node.

When the *Sink* moves along the certain orbit, it broadcasts periodically its current positions, as shown in Figure 2. Thus, the sensor nodes can achieve the current position of the *Sink* in real time. When a sensor node needs to send data to the *Sink*, it can calculate the *θ* following by the mode in Figure 1.

### 2.2. Selecting Next Hop Model Based on Physarum

In this section, we draw a selecting next hop model (SNH) based on physarum foraging mechanism. From paper [18], the flux through each plasmodial tube is as follows:

(3) $$\begin{array}{c} Q_{ij}=\frac{\pi r_{ij}^{4}\left(P_{i}-P_{j}\right)}{8\eta L_{ij}}=\frac{D_{ij}\left(P_{i}-P_{j}\right)}{L_{ij}}=\frac{D_{ij}\Delta P_{ij}}{L_{ij}}, \end{array}$$

where Δ*P*_*ij*_ = *P*_*i*_ − *P*_*j*_ is the difference of pressures, *η* is the viscosity of the fluid, and *D*_*ij*_ = *πr*_*ij*_^4^/8*η* is a measure of the conductivity of the tube.

Physarum forages for distributed food sources through adapting the adaptive behavior of the plasmodium. Consider

(4) $$\begin{array}{c} \frac{d}{dt}D_{ij}=\varphi \left(\left|Q_{ij}\right|\right)-\delta D_{ij}, \end{array}$$

where *δ* is a decay rate of the tube and *φ*(·) is a monotonically increasing continuous function satisfying *φ*(0) = 0. Since (3) and (4) come from fluid dynamics and cannot be directly used in WSNs, we should discuss how to migrate (3) and (4) to WSNs.

Firstly, we discuss the replacement of physical quantities in (3). Because the *D*_*ij*_ is an inherent characteristic of the tube, we replace the *D*_*ij*_ by an inherent physical quantity of wireless link-link quality Φ_*ij*_. Since the meaning of *L*_*ij*_ is the same as that in fluid dynamics, its meaning remains in our model. The replacement of Δ*P*_*ij*_ is rather complex. If flux (e.g., fluid or data packet) wishes migrate from source to destination passing by two other nodes, the fluid tends to flow through the node with lower pressure in fluid dynamics, while data packet should be relayed by the node with higher energy residue and lower angle of deviation. Therefore, we replace the *P*_*j*_ of node *j* by *k* · ER_*j*_ + (1 − *k*)cos⁡*θ*_*jid*_, so does *P*_*i*_ of node *i*. Since *P*_*i*_ is the base pressure, we replace Δ*P*_*ij*_ by *P*_*j*_ through omitting *P*_*i*_. Using (3), we have

(5) $$\begin{array}{c} Q_{ij}=\frac{D_{ij}\cdot \Delta P}{L_{ij}}=\frac{\Phi_{ij}\left[k\cdot {\text{ER}}_{j}+\left(1-k\right)\cos\theta_{jid}\right]}{L_{ij}}, \end{array}$$

where *Q*_*ij*_ is the virtual flux of communication through the wireless link *ij*; Φ_*ij*_ is the link quality; ER_*j*_ is the energy residue of node *j*; *L*_*ij*_ is the Euclidean distance of nodes *i* and *j*; *θ*_*jid*_ is the angle of deviation and its range is [−*π*/2, *π*/2]; *k* is a proportional coefficient which is used to adjust the weight of ER_*j*_ and cos⁡*θ*_*jid*_.

Because of the same characteristic of each node, we suppose that the Φ_*ij*_ of each link is the same and ignore it to simplify discussion. Thus, we have

(6) $$\begin{array}{c} Q_{ij}=\frac{D_{ij}\cdot \Delta P}{L_{ij}}=\frac{k\cdot {\text{ER}}_{j}+\left(1-k\right)\cos\theta_{jid}}{L_{ij}}. \end{array}$$

**Figure 3:**
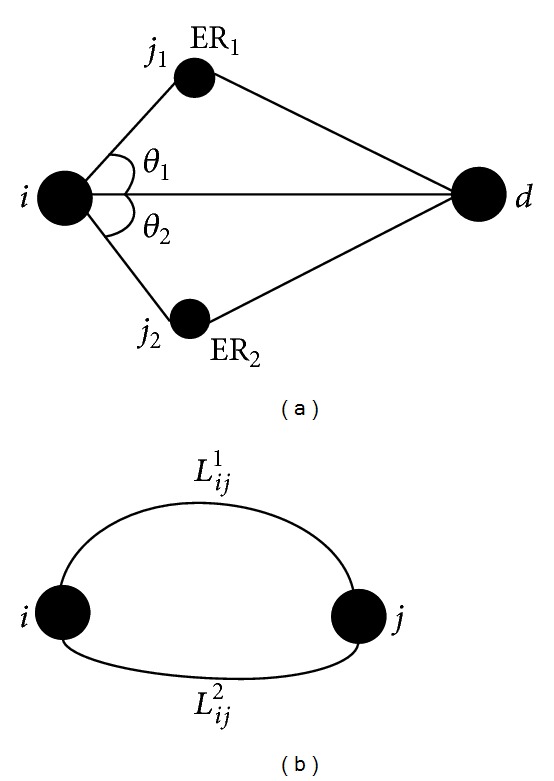
(a) If there are two candidates for next hop, the node who has the greater *k* × ER + (1 − *k*)cos⁡*θ* will be picked as the next hop. (b) If two food sources are connected by two tubes, the longer tube *L*_*ij*_^1^ will vanish with time going by.

Secondly, we analyze the adaptive behavior of plasmodium referring to Figure 3(b), where two food sources are connected by two tubes. Because Δ*P*_*ij*_^1^ = Δ*P*_*ij*_^2^ and *L*_*ij*_^1^ > *L*_*ij*_^2^, the flux *Q*_*ij*_^2^ will be greater than *Q*_*ij*_^1^ from (3). Note that *L*_*ij*_^1^ and *L*_*ij*_^2^ are kept constant throughout the adaptation process in contrast to *D*_*ij*_. Therefore, the adaptive behavior of plasmodium is fulfilled by the evolution of *D*_*ij*_(*t*). In WSNs, node *i* chooses the next hop form candidates as shown in Figure 3(a). Because (1)  *L*_*ij*_1__ and *L*_*ij*_2__ are kept constant, and (2)  Δ*P*_*ij*_1__ and Δ*P*_*ij*_2__ are different and time-varying, we can achieve the adaptation by the evolution of Δ*P*_*ij*_(*t*). Supposing that *φ*(*Q*) = *Q* ^*μ*^, we have

(7) $$\begin{array}{c} \frac{d}{dt}\Delta P_{ij}=\varphi \left(\left|Q\right|\right)-\delta \Delta P_{ij}={\left(\frac{\Phi_{ij}\Delta P_{ij}}{L_{ij}}\right)}^{\mu }-\delta \Delta P_{ij}, \end{array}$$

where *δ* is a decay rate of Δ*P*_*ij*_ and *μ* is a constant satisfying *μ* > 0. We call (7) SNH and use it to determine the next hop in our P-bRS; namely, we choose the node with the largest *d*Δ*P*_*ij*_/*dt* as the next hop.

## 3. P-bRS Routing Scheme

### 3.1. Data Structure in P-bRS

In this section, we introduce the data which should be conserved in each node. Each node *i* needs to conserve the following information: *L*_*ij*_  (*j* ∈ *N*_*i*_), *θ*_*jid*_  (*j* ∈ *N*_*i*_^*R*^), ER_*ij*_  (*j* ∈ *N*_*i*_), ER_*ii*_, and *β*_*ipj*_  (*j* ∈ *N*_*i*_^*L*^), where node *p* is the previous hop of node *i*, ER_*ii*_ represents the ER_*i*_ stored in node *i*, and ER_*ij*_ represents the ER_*j*_ stored in node *i*. As our WSNs are location-aware, the *L*_*ij*_, *θ*_*jid*_, and *θ*_*ipj*_ are easily acquired following (1) and (2). Note that the nodes in our WSNs are fixed, and we only need to calculate the *L*_*ij*_ once at WSNs deployment time. For the difference between ER_*j*_ and cos⁡*θ*_*jid*_, we normalize ER_*j*_ to ${\hat{\text{ER}}}_{j}$. Therefore, we obtain

(8) $$\begin{array}{c} Q_{ij}=\frac{k\times {\hat{\text{ER}}}_{j}+\left(1-k\right)\cos\theta_{jid}}{L_{ij}}, \end{array}$$

(9) ddtΔPij=(k×ER^j+(1−k)cos⁡θjidLij)μ −δ[k×ER^j+(1−k)cos⁡θjid].

### 3.2. Routing Scheme Algorithm

If node *s* needs to send data to the *Sink* *d*, it searches for a routing in the following method.

**Step 1**

Receive the positioning information of the *Sink* node, and then calculate each *θ*_*jid*_ and *β*_*ipj*_.

**Step 2**

Divide each node *j* ∈ *N*_*s*_ into *j* ∈ *N*_*s*_^*F*^ or *j* ∈ *N*_*s*_^*N*^ based on the positioning information of the *Sink* node.

**Step 3**

Calculate each *d*Δ*P*_*sj*_/*dt* (*j* ∈ *N*_*s*_^*N*^) following from (9), where *θ*_*jsd*_, ${\hat{\text{ER}}}_{sj}$, and ${\hat{L}}_{sj}$ are acquired by node *s* beforehand.

**Step 4**

Each node *j* ∈ *N*_*s*_^*N*^ is saved into a temporary array variable *Temp* in descending order by *d*Δ*P*_*sj*_/*dt*.

**Step 5**

The first node in *Temp* is picked as the next hop of the routing.

**Step 6**

If the next hop *a* of node *s* satisfies *N*_*a*_^*N*^ = *∅*, namely, there is an energy hole in WSNs, the node *a* will not send *ACK* to *s*. Then, the node *s* will trigger a specific processing routine.

**Step 7**

If |*Temp*[0] → *θ*_*jsd*_ − *Temp*[1] → *θ*_*jsd*_ | ≥*π*/2, node *s* will choose the node *Temp*[1] as the next hop. Then, the regular processing routine is going on.

**Step 8**

Otherwise, each *d*Δ*P*/*dt*  (*j* ∈ *N*_*s*_^*F*^) is calculated following (10) and the nodes are saved into the *Temp* in ascending order by *d*Δ*P*/*dt*. Then, the first node in *Temp* is chosen as the next hop of the routing and the regular processing routine is going on. Consider

(10) ddtΔPij=(k×ER^j+(1−k)βasjLij)μ −δ[k×ER^j+(1−k)βasj],

where *β*_*asj*_ is the angle of line *sa* and line *sj*. Equation (10) indicates that it tends to choose a node which sharply deviates from the node *a* as the next hop to avoid entering the energy hole again.

**Step 9**

The process is repeated, like a rolling wheel, until the *Sink* *d* is found.

### 3.3. Algorithm Analysis

In this section, we analyze the feasibility of SNH by mathematical theoretical analysis. We study cases in which two nodes connected to the same node compete to be the next hop, as shown in Figure 3(a).

There are four nodes *i*, *j*_1_, *j*_2_, and *Sink* *d*. For simplicity, we hereafter replace *L*_*ij*_1__, *L*_*ij*_2__, *Q*_*ij*_1__, *Q*_*ij*_2__, Δ*P*_*ij*_1__, and Δ*P*_*ij*_2__ by *L*_1_, *L*_2_, *Q*_1_, *Q*_2_, Δ*P*_1_, and Δ*P*_2_, respectively. In multipath routing, the virtual fluxes along each path are calculated as follows:

(11) $$\begin{array}{c} Q_{1}=\frac{\Delta P_{1}/L_{1}}{\Delta P_{1}/L_{1}+\Delta P_{2}/L_{2}},\\ Q_{2}=\frac{\Delta P_{2}/L_{2}}{\Delta P_{1}/L_{1}+\Delta P_{2}/L_{2}}. \end{array}$$

Since *Q*_1_ and *Q*_2_ are nonnegative, adaptation equation (7) becomes

(12) $$\begin{array}{c} \frac{d}{dt}\left(\Delta P_{1}\right)=\varphi \left(Q_{1}\right)-\delta \cdot \Delta P_{1},\\ \frac{d}{dt}\left(\Delta P_{2}\right)=\varphi \left(Q_{2}\right)-\delta \cdot \Delta P_{2}. \end{array}$$

Setting *φ*(*Q*) = *Q* ^*μ*^, (*d*/*dt*)(Δ*P*_1_) = 0, and (*d*/*dt*)(Δ*P*_2_) = 0, we have

(13) $$\begin{array}{c} {\left(\frac{\Delta P_{1}/L_{1}}{\Delta P_{1}/L_{1}+\Delta P_{2}/L_{2}}\right)}^{\mu }=\delta \cdot \Delta P_{1},\\ {\left(\frac{\Delta P_{2}/L_{2}}{\Delta P_{1}/L_{1}+\Delta P_{2}/L_{2}}\right)}^{\mu }=\delta \cdot \Delta P_{2}. \end{array}$$

From (13), we obtain

(14) $$\begin{array}{c} \Delta P_{2}=\left(\delta^{-1/\mu }{\left(\Delta P_{1}\right)}^{1-1/\mu }-\Delta P_{1}\right)\frac{L_{2}}{L_{1}}. \end{array}$$

From (13) and (14), we obtain

(15) $$\begin{array}{c} \Delta P_{1}=\frac{1}{\delta }{\left[\frac{1}{\left(1+{\left(L_{1}/L_{2}\right)}^{1/1-\mu }\right)}\right]}^{\mu }. \end{array}$$

Similarly,

(16) $$\begin{array}{c} \Delta P_{2}=\frac{1}{\delta }{\left[\frac{1}{\left(1+{\left(L_{2}/L_{1}\right)}^{1/1-\mu }\right)}\right]}^{\mu }. \end{array}$$

**Figure 4:**
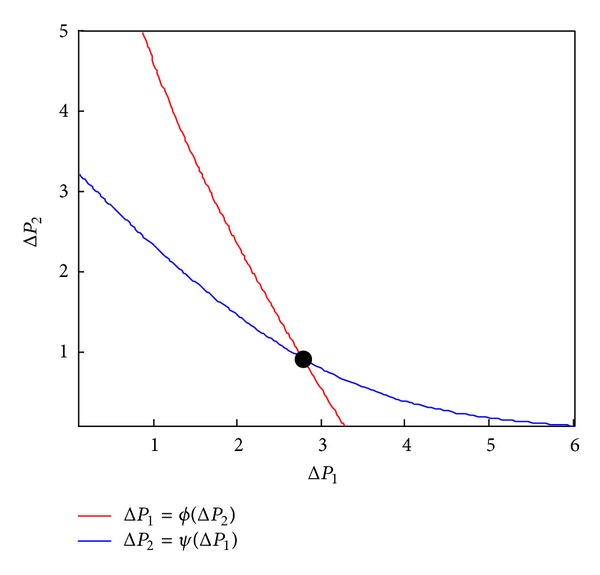
Asymptotic behavior of the solution in *μ* = 0.8, *δ* = 0.3, *L*_1_ = 10, and *L*_2_ = 12: two curves which come from (13) intersect in a point *E*, which is the sole equilibrium point.

Namely, equilibrium point is given by (Δ*P*_1_, Δ*P*_2_). We perform the simulation by setting the parameters *μ* = 0.8, *δ* = 0.3, *L*_1_ = 10, and *L*_2_ = 12 following from (13), and the visualization of the solutions is shown in Figure 4, where point *E* is the equilibrium point of two curves. Therefore, the routing of WSNs will reach equilibrium with our SNH, which is very important to a routing strategy.

## 4. Simulation Results

**Figure 5:**
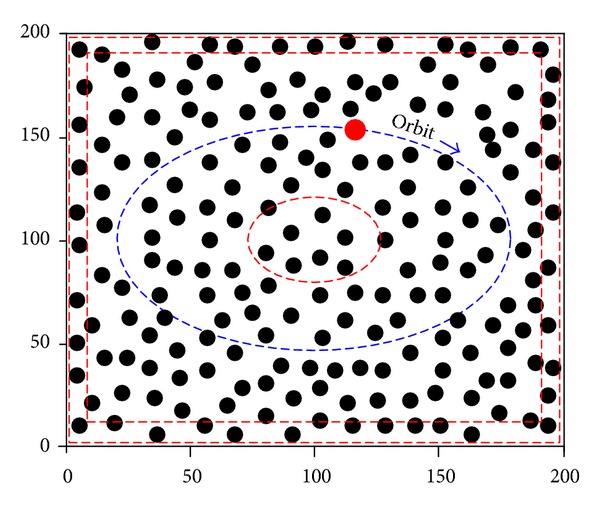
Sensors distribution.

We design a simulation platform using C++ to validate P-bRS. In the simulation, 400 sensors are relatively regularly deployed in the field of 200 m × 200 m, and the *Sink* node is deployed the right of the field, as shown in Figure 5. The sensing radius of each sensor is 30 m, the original energy of each node is 100, and the energy of the *Sink* node is inexhaustible. We suppose that the energy consumption of one transmission is 1, if the transmission distance is 20 m. Therefore, the energy consumption of one transmission of two nodes *i* and *j* is (*L*_*ij*_/20).

In order to validate the energy equilibrium, we only choose the nodes in the center or peripheral simulation field (enclosed by red dashed circle or two red dashed rectangles in Figure 5) to transmit data to the *Sink*. If a chosen node transmits a group of data to the *Sink*, the P-bRS is used to choose next hops until the *Sink* is found, which is called a *round*. This iterative process will halt after *n* rounds until WSNs break down. We run GPSR, GEAR (*k* = 0.5, 0.9, where we use *k* to replace *α* which is used in [24] to bring into correspondence with P-BRS) and P-bRS (*k* = 0.5, 0.9) 10 times, respectively, to acquire their average value and compare them. If the distance between the nodes and the *Sink* is less than 30 m, we set the nodes to directly transmit data to the *Sink* to quicken convergence of P-bRS, and the energy consumption is set to 1.

### 4.1. Energy Equilibrium of P-bRS

**Figure 6:**
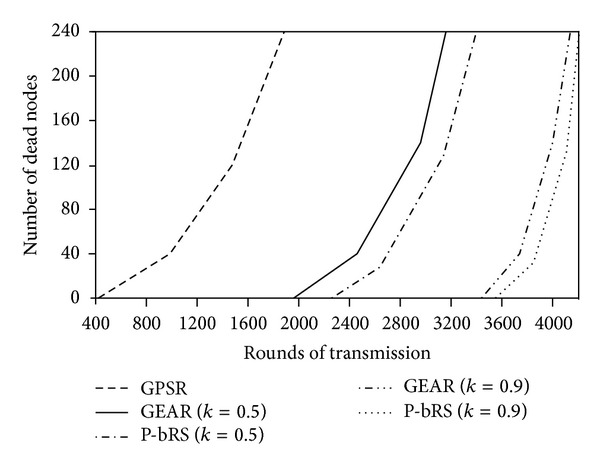
Lifetime of network.

Figure 6 illustrates the lifetime of WSNs. In GPSR, the first dead node emerges in round of 406, and the WSNs break down in round of 1857. In GEAR (*k* = 0.5), the first dead node emerges in round of 1955, and the WSNs break down in round of 3042. In P-bRS (*k* = 0.5), the first dead node emerges in round of 2284, and the WSNs break down in round of 3351. In GEAR (*k* = 0.9), the first dead node emerges in round of 3462, and the WSNs break down in round of 4043. In P-bRS (*k* = 0.9), the first dead node emerges in round of 3524, and the WSNs break down in round of 4108. Therefore, the lifetime of GEAR (*k* = 0.5) is 63.8% longer than that of GPSR; the lifetime of P-bRS (*k* = 0.5) is 10.2% longer than that of GEAR (*k* = 0.5); and the lifetime of P-bRS (*k* = 0.9) is 1.6% longer than that of GEAR (*k* = 0.9). From Figure 6, we can differ that (1) whether considering energy residue of next hops or not will impact on the lifetime of WSNs greatly; (2) in energy balanced WSNs, the time period is very short from emerging dead nodes to networks breaking down, because all nodes reach exhausted status of energy in same time period.

**Figure 7:**
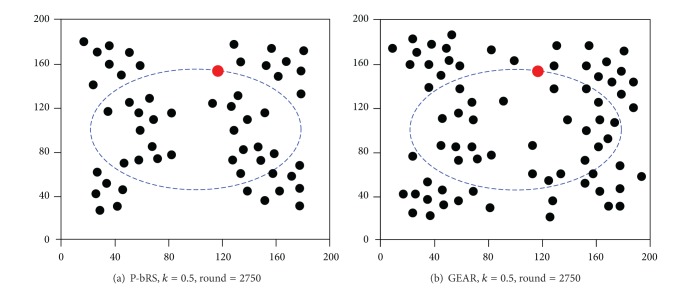
Distribution of dead sensor nodes.

Figure 7 illustrates the dead nodes distributions of GEAR (*k* = 0.5) and P-bRS (*k* = 0.5) in the rounds of 2750. The results show that P-bRS (*k* = 0.5) has much less dead nodes than GEAR (*k* = 0.5). We can also differ that the dead nodes of both algorithms are converged on a specific field but not spread around the entire range of WSNs, which is useful in deploying such WSNs to prolong the lifetime through rationally deploying the specific field.

### 4.2. Efficiency of P-bRS

**Figure 8:**
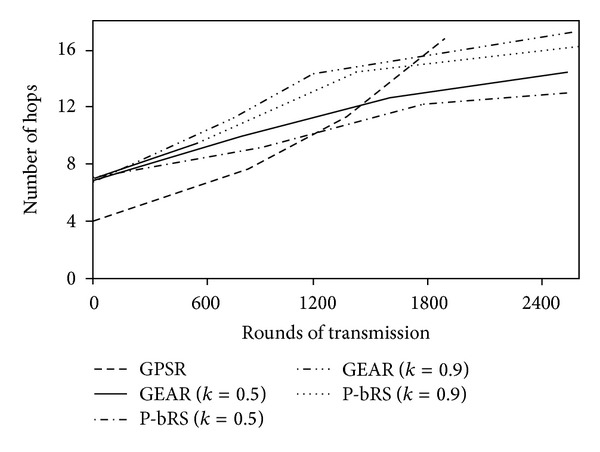
Needing hops of transmission of each round.

Figure 8 illustrates the number of hops that the different algorithms need in different rounds of transmission. By calculating, the average hops of GPSR, GEAR (*k* = 0.5), P-bRS (*k* = 0.5), GEAR (*k* = 0.9), and P-bRS (*k* = 0.9), are 9.7, 12.2, 11.1, 14.8, and 13.3, respectively.

In the case of *k* = 0.5, the average hop of P-bRS is 14.4% more than that of GPSR, and the hop of GEAR is 25.8% more than that of GPSR. Combing with Figure 8, the increment of average hops of 14.4% will lead to the increment of lifetime of more than 70% from GPSR to P-bRS, while the increment of average hops of 25.8% will only lead to the increment of lifetime of more than 60% from GPSR to GEAR. Therefore, the P-bRS is more efficient in balance of routing efficiency and energy equilibrium than GEAR.

In the case of *k* = 0.9, the average hop of P-bRS is 37.1% more than that of GPSR, and the hop of GEAR is 52.6% more than that of GPSR. Combing with Figure 6, the increment of 14.4% of average hops will lead to the increment of about 70% of lifetime from GPSR to P-bRS (*k* = 0.5), while the increment of 19.8% of average hops will only cause the increment of 22.6% of lifetime from P-bRS (*k* = 0.5) to P-bRS (*k* = 0.9). That is to say, the larger *k* is, the smaller the increment of *k* impacts on lifetime of WSNs. Therefore, it is improper to set a larger *k*, so does GEAR.

## 5. Conclusion

The physarum forages for patchily distributed food sources through accommodating its body to form networks with comparable efficiency, fault tolerance, and cost. We draw inspiration from the physarum model and improve it to suit the routing choice for WSNs. The P-bRS algorithm can deal with the trade-off between routing efficiency and energy equilibrium in WSNs, which greatly reduces the processing delay and saves the energy of sensors. Based on the simulation results, we discuss the P-bRS's performance. In future work, we consider introducing actual mobility model of nodes into P-bRS to make it fit in with mobile WSNs. Moreover, we consider the model may also provide a useful help to develop the routing protocols in other networks, which will be our future focus.
